# Pre-service Teachers’ Reflections on Attitudes Towards Teaching and Learning Mathematics with Online Platforms at School: A Case Study in the Context of a University Online Training

**DOI:** 10.1007/s10758-022-09602-0

**Published:** 2022-04-25

**Authors:** Frederik Dilling, Amelie Vogler

**Affiliations:** University of Siegen, Herrengarten 3, 57072 Siegen, Germany

**Keywords:** Attitudes of pre-service teachers, Case Study, Online learning platforms, Qualitative content analysis, Teacher education, Technology in mathematics education

## Abstract

Online learning platforms take over a new role in education, especially in times of the Covid-19 pandemic. This paper will discuss pre-service teachers’ reflections on attitudes towards online learning platforms and the respective changes due to an online training of using this digital tool in mathematics classes. The special training took place in a bachelor seminar with fourteen participants on the use of digital media in mathematics education at the University of Siegen. Based on the ‘Tripartite Model of Attitude Structure’ which defines the psychological concept of attitude, data material about the pre-service teachers’ attitudes is gathered by pre- and post-reflection-questionnaires. A qualitative content analysis led to the formation of a system of six main categories and approximately 51 descriptors for pre-service teachers’ attitudes towards online learning platforms and especially their changes due to the online training of using these platforms. The descriptors can be a basis for further research studies on this topic.

## Introduction

The global crisis caused by the spread of Covid-19 takes *distance-learning* to a new level of importance in education. The educational system is faced with the unavoidable task of enabling students to learn through the use of digital media. Engelbrecht et al. (2020) reported about teachers’ first experiences during their first months of distance-learning:

*Teachers’ brief has changed overnight. They had to jump in at the deep end—sometimes with little advance notice and often with little support. As schools were closing some teachers frantically had to gather materials to use online. […] Teachers encountered new problems and can feel isolated and uncomfortable in this environment. They had to spend days and weeks constructing new materials in new formats for online instruction. They formed support groups, shared materials and created new innovative approaches in some cases. They spent huge amounts of time answering questions, working with students individually or in groups, and communicating with concerned parents.* (p.823f)

On the one hand, working with new technologies seems to offer new opportunities and possibilities, especially in distance-learning situations. On the other hand, it poses enormous challenges in terms of content, organization, and technology for all people working in the educational sector. Findings of the survey on participants’ perceptions of the teaching workforce’s preparedness to teach mathematics online (Clark-Wilson et al., 2020, p. 1236) show that there are differences between countries and even regions regarding the preparedness to teach mathematics online. For example, Italian and Turkish secondary teachers stated to be highly prepared in contrast to French, Mexican and Netherlands teachers who felt to be more unprepared to teach mathematics in an online format. One reason could be that in Turkey, the Ministry of Education has already started to design an online teaching platform in 2012. Therefore, when schools closed in 2020, mathematics teachers were able to access and offer mathematical content to their students that included lesson videos, summaries of the units, exercises and tests to all students at any time, while in other countries like Mexico, teachers and students exchanged activity sheets by taking and sending photos on mobile phones or via home visits (ibid.). These differences could be explained by ministerial guidelines, local access to the internet and other technological resources and teachers’ confidence to use videoconference tools for synchronous communication with their students (Drijvers, 2020).

There are first studies that have examined (possible) effects of school closures on the performance of the students. The data collected in the first months of the closures predicts that the lockdown due to Covid-19 could have a high influence on the students’ learning processes of grades 3 to 8 in comparison to a typical school year, especially in mathematics: “Students are likely to show much smaller learning gains, returning with less than 50% of the learning gains and in some grades, nearly a full year behind what we would observe in normal conditions” (Kuhfeld & Tarasawa, 2020, p. 2). Also other empirical studies explored the specifics of online learning (e.g. Ehsanpur & Razavi, 2020; Razavi, 2013).

A detailed review (cf. Trenholm & Peschke, 2020) shows the differences between teaching in fully online and face-to-face modalities at University. The authors stated that an online course is accessed through an institutional learning management system (e.g. Moodle) which we will also describe in Sect. 2.1 after defining distance-learning and blended-learning as concepts of online learning. Trenholm and Peschke conclude that “exploiting the potential of online education takes work, ingenuity, inventiveness, a willingness to experiment with new technology, and a faith in your own abilities to teach—all with an attentive ear to your students’ opinions” (Trenholm & Peschke, 2020, p. 15).

Although there are already some empirical studies on online learning platforms (see Sect. 2.2), there is still a large research gap regarding the interaction of teachers with these systems. In particular, there are questions about the importance of teachers' attitudes in this context and how these attitudes are shaped. The empirical study in this paper is intended to provide initial findings in this regard with a focus on the integration and implementation of online learning platforms in mathematics education in high school. Therefore, in Sect. 2.2, we will present the state of research on the use of online learning platforms such as Moodle in mathematics education at school. These online platforms provide a choice of many different tools, for example the inclusion of text, graphics and video casts as well as access to outside resources (via web links), inbuilt communication channels (e.g. discussion forums), interactive scheduling of activities, and drop boxes for assignments (ibid.). Obviously, the teacher and his or her attitudes are important factors for the use of these multifunctional learning platforms, as they are responsible for the design, structure, and content of the platform used during a distance-learning period, which is currently a standard format of teaching due to the pandemic. Motivated by these facts and circumstances, empirical evidence will be provided for pre-service teachers’ attitudes towards the use of online learning platforms for high school mathematics and especially their changes due to a specifically designed online training. The focus is on the following research question:

*How do pre-service teachers reflect their attitudes towards online learning platforms in mathematics class at school and how do their reflections change after an online training course on this topic?*

This question will be investigated by a research design consisting of two open reflection questionnaires (pre and post) completed by fourteen participants of an online seminar for pre-service teachers in their bachelor’s program of mathematics teacher education at university. The *Tripartite Model of Attitude Structure* (Breckler, 1984), which defines the psychological concept of attitude regarding three components (affect, behavior, and cognition), is described in detail in Sect. 3.2 and used as a theoretical basis for the data collection and analysis (Sects. 4.1 and 4.2) of pre-service teachers’ attitudes. Using the method of qualitative content analysis according to Mayring (2000), a system of six main categories and 51 descriptors for the pre-service teachers’ attitudes towards online platforms and their changes due to the training of using the online learning platform Moodle are identified.

## Online Learning Platforms in Mathematics Education

### Online Learning Platforms in the Context of Distance-Learning and Blended-Learning

*[O]nline Learning [is] becoming a more viable and attractive option for students and teachers around the world.* (Avineri et al., 2018, p. 187)

In this context, the following concepts can be distinguished: *distance-learning* and *blended-learning*. In distance-learning formats (also called e-learning), all teaching and learning events take place online. In contrast, blended-learning describes the alternation of online and presence phases. Since the beginning of the twenty-first century, the concept of blended-learning has been regarded as a new culture of teaching and learning and forms a new line of research in education (Petko, 2010a). It enables the connection of teacher inputs, independent work phases, individual activities, group phases, traditional texts, multimedia learning materials, and traditional as well as new elements. The main instruments of this learning setting are the computer and the internet. According to Jonasson (2000), digital media can be seen as *cognitive tools* for the distribution of knowledge because they structure the information processing (e.g. in mind maps or databases) and enable reflection (e.g. through e-portfolios). Taking another Look at the current education situation in the context of the pandemic, blended and online learning has developed from “*important* to *essential*” (Engelbrecht et al., 2020a, b, p. 836).

The blended-learning concept also offers a variety of possibilities for classrooms such as the support of didactic arrangements through multimedia illustrative material, the publication of learning outcomes, collaboration beyond the classroom (e.g. cooperation on homework, exchange of resources among teachers), and much more. Considering these aspects, it is easy to see *that (online) learning platforms* can fulfill a central role in the teaching–learning process. Considering the transformation that learning is seen as an active, self-controlled, constructive, emotional, situational, and social process (Reinmann-Rothmeier & Mandl, 2006), teachers act less as knowledge transmitters and more as *learning facilitators* and role models in this teaching–learning concept. An indispensable postulate, which applies to distance-learning and blended-learning is that it must not be a matter of adapting didactics to the possibilities offered by technology, but rather the other way round (Pekto 2010a). Pérez-Álvarez et al. (2018) describe tools to support self-regulated learning in online environments in a literature review. Self-regulated learning can be defined as “the ability to plan, monitor, and actively control one’s learning process (Davis et al., 2018, p. 122). Especially in MOOCs (Massive Open Online Courses, a sort of online learning platform), self-regulated learning skills are important because teacher guidance is rare and learners must engage in their learning process trying to succeed and achieve the learning goals (Pérez-Álvarez et al., 2018). The original vision of MOOCs is to make higher education accessible to those that do not enter the traditional tertiary education system (Davis et al., 2018).

The offer of online learning platforms differs in its functional range and structure but the following common feature should be noted: “Learning platforms are programs which are operating on an internet server and whose function can be accessed by users via a standard internet browser” (Petko, 2010a, p. 17, author’s translation). A well-known open-source software, to which the following study refers, is *Moodle* (https://moodle.org/). Learning platforms comprise different modules, for the creation and management of content (*learning content management systems—LMS*) on the one hand and for the composition and timing of this content (*course management systems*) on the other. Clark-Wilson et al. (2020) describe that MOOCs can serve as an infrastructure for communication and representation infrastructure that offers a virtual environment for the activation of new connections between colleagues as well as access to resources. Referring to an Italian study about mathematics teachers’ networks, the authors explain that working and communicating in such a digital environment enables the teachers to implement classroom activities using technologies such as GeoGebra.

Since June 2020, all schools in Germany, especially in North-Rhine-Westphalia, have the possibility to use the Moodle-based LMS *LOGINEO NRW* (https://www.logineo.schulministerium.nrw.de/) free of charge. Therefore, it seems effective to use Moodle as an example of a learning platform in the online training course for pre-service teachers of the following study. In general, the main aspects or the structure of a learning platform can be described as follows (cf. Petko, 2010a):

- Personal identification by password
- Learners, instructors, and administrators have different user interfaces and therefore different rights
- Learners have access to an individual selection of learning content, tasks, tests, and communication functions
- Teachers have access to further functions such as writing announcements or calling up user statistics

The most important functional features (cf. Petko, 2010a, b) of the learning platform *Moodle* are the following:

- *Content Feature* Creation and password protected display of sequenced learning content in the form of multimedia files, import and export of files, podcasts, etc.
- *Communication Feature* Announcement function, email, forums, blogs, chat, audio or video conferencing
- *Task and Timeline Feature* Calendar, scheduled learning tasks, scheduled display and hiding of specific functions, and submission function
- *Test and Survey Function* Online tests with automated evaluation and immediate feedback, surveys with automated counting, and status reports for task completion
- *Administration Feature* User administration, course administration, allocation of roles and rights, statistics and monitoring function

According to Paechter et al. (2013), decisive advantages of such a learning platform in comparison to face-to-face-activities are the fast exchange of information, the storage of files in folder-like form, the long-term access to files, the sending of information to individuals or groups, and the generation of automatic notifications (e.g. “Upcoming events”). Overall, a learning platform can be considered an extended infrastructure of a school (Petko & Moser, 2010).

### Use of Online Platforms in (Mathematics) Education in School

In this subchapter, we would like to focus on the use of learning platforms in the field of (mathematics) education in school.

Henríquez et al. (2018) explored the impact of the use of *Khan Academy* platforms as instructional and practical teaching material in primary and secondary school classrooms in Chile. This platform is an online platform allowing free and unlimited access to academic content validated by professors specialized in mathematics or other disciplines. The principal benefits for the teaching and learning process are the following (ibid.). Classes become more dynamic because of the use of instructional videos. Students have access to teaching materials at home, so they can obtain good results in class contents at home. Teachers can keep better track of their students’ progress which allows them to give personalized attention to the diversity of students in the classroom because of the constant assessment and feedback provided by the platform. The teacher has a dominant role in the adoption of the platform and it cannot be used effectively without a guide. The platform promotes autonomous learning and self-efficacy in order to increase student participation and motivation in learning mathematics.

According to Pérez-Álvarez et al. (2018), the use-visualization and the interactive elements have a positive effect on learners’ motivation. They refer to the Chilean *Coursera* platform that offers the option of consulting the time spent on video lessons and has a submission timetable and email notifications that help learners to keep engaged with the course. Furthermore, Davis et al. (2018) write that learners need tools that enable them to learn how to learn. They refer to the Netherlands *edx* platform “that allows learners to explicitly *express* their motivation, *plan* their learning, *monitor* their progress towards their set goals at any point in time, and *reflect* on them” (p. 123). Referring to a study of Zhao et al. (2018) on the edx platform, MOOCs allow both, *mobile learning* and *stationary learning*. The former is to be understood as any sort of learning that happens when the learner is not at a fixed, predetermined location and as learning that happens when the learner takes advantage of the learning opportunities offered by mobile devices. The latter is to be understood as a (common) learning situation in which learners use a device with a large screen to access course materials whilst being stationary in a comfortable environment which enables them to focus on the learning activity.

Regarding collaboration and communication processes in distance-learning compared to face-to-face-learning, we refer to the investigation of Paechter et al. (2013). They analyzed the experiences of Austrian students and their preferences for online- or face-to-face-communication. *Online-communication* is preferred because of various possibilities for establishing contact with teachers and the fast exchange of information. *Face-to-face-communication* is preferred because of the development of socio-emotional relationships with the teacher. Overall, students prefer a rich communication scenario but learning platforms are often not fully exploited and only text-based communication is used instead of video conferencing. More recent research by Drijvers and several other researchers from Germany, Netherlands, and Belgium, in 2020, confirms that even during the phase of online learning due to the Covid-19 pandemic, nearly a third of the German teachers surveyed has never used a synchronous format such as video conferencing and 27% of the teachers have used a synchronous format once a week. And only about a fifth of the teachers has used it multiple times a week. As a comparison, in the Netherlands, about 66% have used it multiple times a week.

Empirical findings on the use of learning platforms show that these are relevant for learner-centered teaching and cooperation with external partners. Despite, the use of learning platforms in school is often limited to the email function and providing files, mostly in lists of links. Findings from the Swiss *good-practice-studies* of Petko (2010b) show that the learning platform primarily serves as a distribution tool for the teacher and less as a tool that students can use to participate and communicate. Results of Moser and Petko (2010a, b) confirm that school management plays a key role in the implementation of the learning platforms. Also, the competences and attitudes of the teachers and their use of training opportunities have a significant impact on the use of learning platforms. In the following chapter 3, we define the term attitude (Sects. 3.1 and 3.2) and explain attitudes towards the use of technology in mathematics education (Sect. 3.3) that provides and expands the theoretical background of our study described in chapter 4.

## The Theoretical Framework of Attitudes

### The Definition of Attitude

*Attitude* is a fundamental term from psychology, but in literature, there are various definitions of the term *attitude*. For example, according to Auzmendi (1992) attitudes are aspects that are not directly observable but defined as comprised of both, beliefs and behavioral predispositions towards the targeted object. Besides, Gómez-Chacón (2000) defines attitude as evaluative bias (negative or positive) that determines the personal and behavioral intention. Gal and Ginsburg (1994) summarize that in the context of education, attitude is the sum of all the emotions and feelings experienced during the learning phase of the studied subject. According to the research of Gomez-Chacon (2000), attitude is a main element in the teaching–learning process because its development is important for the teaching and learning of any subject.

The term attitude is also frequently used in the research of mathematics education. Di Martino (2016, p. 4) states that there are two recurrent definitions of attitude:

1. Attitudes are positive or negative feelings associated with math. *(simple definition)*
2. Attitude is a construct of three components: the emotional disposition, the set of beliefs regarding mathematics, and the behavior related to mathematics. *(three-dimensional definition)*

Another interesting aspect in this context is the understanding of Ruffel et al. (1998) who state: “We conjecture that perhaps it [attitude] is not a quality of an individual but rather a construct of an observer’s desire to formulate a story to account for observation” (p. 1).

### The Psychological Concept of Attitudes

**Fig. 1:**
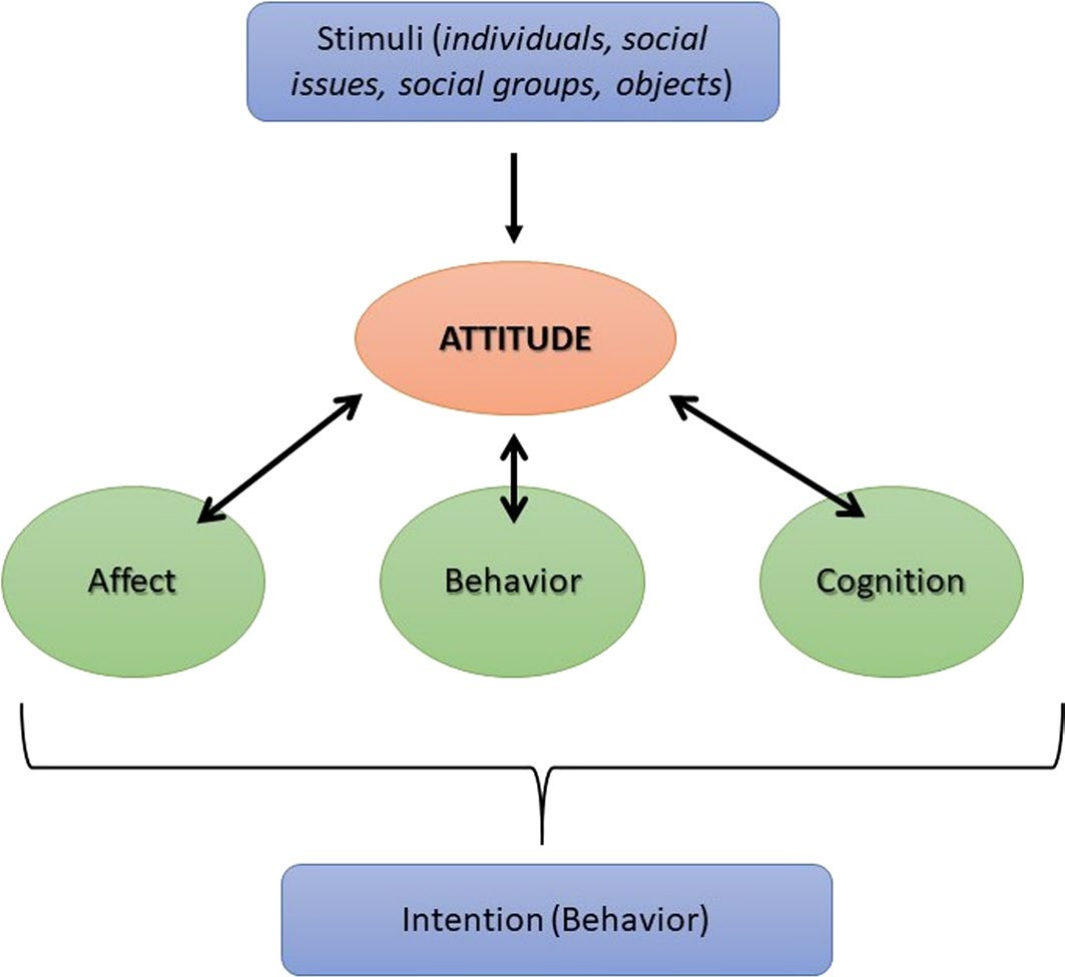
The ABC-model of attitudes

In our case study, attitudes provide an interpretative instrument to understand the reasons for intentional actions that involve complex relationships between affective and cognitive aspects. Attitude is a psychological construct of multiple factors, which can be measured as dimensions of attributes. This evaluative quality distinguishes the concept from beliefs or opinions. In the following, we refer to the *Tripartite Model of Attitude Structure* described in Breckler (1984). Within the model (Fig. 1), attitude is defined as a response to a **previous stimulus or attitude object** which is or is not observable. The response to that stimulus is divided into three hypothetical, unobservable classes: *affect, behavior, and cognition*. These classes (also called components or factors) influence one’s **behavioral intention**, which is the immediate motivational factor for the behavior itself (Van Aalderen-Smeets et al., 2012). The described model is also referred to as *ABC-model* in psychology because of the initial letters of affect, behavior, and cognition.

The component ***affect*** refers to an emotional response, reaction, or (sympathetic nervous) activity. It can be measured by monitoring physiological responses or by collecting verbal reports of feelings or moods and it is based on emotional experiences or preferences (positive and negative) (Breckler, 1984). The affectively-based attitude usually comes from a person’s values (e.g. about sensitive issues like politics, sex, and religion) and is used to express and validate its moral belief or value system. Santillan et al. (2012) write that this component collects all those emotions and feelings that stimulate the attitudinal object (e.g. subjective reactions of trust and distrust, like and dislike).

The component ***behavior*** refers to the way one behaves when exposed to an attitude object. According to Eagly and Chaiken (1993), it is based on overt (or covert) actions (or verbal statements regarding behavior) that people exhibit concerning attitude objects. It is related to expressions of behavioral intention or behavior that represent the tendency to act or resolve in a specific way (Santillan et al., 2012). This component is derived from past behavior formed by direct or indirect experiences. People often tend to infer attitudes that are consistent with prior behaviors.

The component ***cognition*** is determined by beliefs, knowledge structures, perceptual responses, and thoughts or thinking skills. According to Eagly and Chaiken (1993), it exists when individuals process information about the attitude object which then forms beliefs. In that sense, attitude is an individual’s belief about whether the outcome of his or her behavior will be positive or negative. Santillan et al. (2012) summarize that this component refers to the mental process of perception, conceptions, and beliefs about the attitudinal object.

### Attitudes Towards the Use of Technology in Mathematics Education

In the following section, we provide an insight into the state of research on attitudes towards the use of technology in (mathematics) education. Technology implementation and integration in schools is an important and frequently discussed topic. One objective is to encourage schools, districts, and states to infuse technology into education and to provide tools and resources in order to develop learning skills and enhance students’ experiences in the classroom. In this process of pedagogical transformation regarding the (effective) use of digital media in teaching, the teacher appears to play a crucial role as the “innovator” (Tondeur et al., 2008). More importantly, educational research shows that both teaching and learning (including the use of technology) are affected by the attitudes of the teacher (e.g. Van Aalderen-Smeets et al., 2012; Nordlöf et al., 2019). This is not surprising, since the teacher is responsible for the design of the classes.

*Barriers to successful technology adoption appear to have internal and external sources. Internal barriers may be summarized as “teacher attitude” or “perceptions” about technology. External sources include the availability and accessibility of necessary hardware and software, the presence of technical personnel and institutional support, and a program for staff development and skill building.* (Rogers, 2000, p. 459)

This quote reveals that in addition to technical and personal resources, teachers’ attitudes are an important factor influencing the use of (new) digital technologies in teaching. A considerable amount of empirical research in this field proves that besides different factors like teachers’ background variables, their pedagogical beliefs, their perceived training effectiveness, and organizational support, *teachers’ attitudes towards technology* (including their perceived beliefs of competences regarding technology, or self-efficacy in using technology) can play an important role in the actual use of technology in school education (e.g. Ertmer, 2005; Ertmer et al., 2012; Drent & Meelisen, 2008). According to Baylor and Ritchie (2002) technology integration is related to teachers’ willingness to change.

*Regardless of the amount of technology or its sophistication, technology will not be used unless faculty members have the skills, knowledge, and attitudes necessary to infuse it into the curriculum.* (ibid., p. 298)

A closer look at the current literature indicates that the attitude of teachers towards concrete devices like laptops (Shain et al., 2016) and mobile devices like tablets in educational contexts (Khlaif, 2018) or towards the use of technology in general (Li et al. 2019; Van Aalderen-Smeets et al. 2012; Nordlöf et al. 2019; Thurm, 2017) has been the main focus of research on attitudes towards the integration of technology in education in recent years.

Results of the study of Sahin et al. (2016) confirm that teacher training on the use of technology in the context of education can be an important factor influencing the attitudes and use of technology in the classroom. They refer to Ropp (1999) who wrote that most of the teacher education programs have a lack of adequate technology-related courses and if teachers do not improve their technology skills, their attitudes towards technology in the classroom might be affected negatively and thus, they might be hesitant to use technology. Sahin et al. (2016) conclude that “it is essential to pilot technology use in teaching with pilot courses before and during the teaching career” (p. 364).

The Study of Nordlöf et al. (2019), who explored teachers’ perceptions of and attitudes towards technology education in Sweden, provides the following results: Self-efficacy mainly comes from experience, education, and interest. In contrast, a negative attitude comes from a lack of support and resources, which impedes the teaching with technology. They conclude that teachers who have been trained and educated in the use of technology generally express more positive attitudes and thus seem to have advantages concerning technology teaching. Nevertheless, these teachers still sometimes express negative attitudes related to their perceived control. According to Li et al. (2019), who investigated high school teachers’ use of technology, the teachers’ technology self-efficacy is a predictor of their use of technology. Technology self-efficacy denotes the teachers’ self-related technology skills as beliefs about themselves. Teachers’ positive attitude towards technology, such as passion about technology, openness towards technology, or the aspect of feeling comfortable with using technology, seems to affect their technology integration practices (Hew & Brush, 2007). However, teachers’ (pre-existing and possibly negative) attitudes can also be a major barrier to the integration of technology (ibid.). A more recent study of Ndlovu et al. (2020) that investigated pre-service teachers’ beliefs about their intentions to integrate information and communication technologies in their future classrooms implies that pre-service teachers need to be exposed to different ways of teaching mathematics using these technologies, both familiar ones like a graphing calculator, GeoGebra, and YouTube videos as well as less familiar ones like CAS and online platforms in order to increase their self-efficacy in many advanced tools, at an early stage of their training program.

This short literature review shows that academic research of the factors influencing the attitude towards the use of technologies in the classroom and academic training of its use can support the process of integration and implementation of technology in classrooms more effectively. This hypothesis should be the basis for the case study presented in the following chapter.

## Case Study

### Methodology and Conditions

The literature review has shown the importance of teachers’ attitudes towards the use of technology for the implementation of (new) digital technology in their classrooms. Several studies have empirically proven this connection and have also described the influence of specific teacher training programs on the use of technology on the teachers’ attitudes. In this article, this connection will be investigated in a case study with a focus on using online learning platforms. The following research question is examined:

*How do pre-service teachers reflect their attitudes towards online learning platforms in mathematics class at school and how do their reflections change after an online training course on this topic?*

The basis of the case study is a training on online learning platforms using the example of *Moodle* for pre-service teachers in mathematics education at the University of Siegen. It took place in May 2020 as part of a seminar on digital media in mathematics education, which is an elective seminar in the bachelor’s program for pre-service teachers. Due to the Covid-19 pandemic, the entire course took place online and consisted of a combination of virtual synchronous meetings and self-study phases. 19 pre-service teachers who were studying mathematics education for high school took part in the seminar. Most of them were in the fifth or sixth semester of their bachelor’s program and almost all of them have only been able to gain teaching experience in the required internships (8 weeks during the bachelor’s program). Furthermore, they mentioned that they have gained some experience with the use of digital media in the classroom in another seminar at university. None of them had experience in distance-learning in school. The learning management system commonly used in teacher education at the University of Siegen is the platform *Moodle*. Hence, the participants of our study were familiar with the basic functions of Moodle, at least from a learner’s perspective.

At the beginning of the training, all pre-service teachers were introduced to the most important functions of *Moodle* in an online meeting and had the opportunity to ask questions. In the subsequent self-learning phase, the pre-service teachers read a text on learning platforms, e-learning, and blended learning at school and answered key questions based on the text. The core of the training consisted of the independent development of an online learning unit with *Moodle* either on the Pythagorean Theorem, the Binomial Formulas, or Derivation. In this learning unit, the possibilities of *Moodle* should be used creatively, but at least the pre-service teachers should upload two files or link two internet pages, create a vote, create a task for submission, upload interactive content (e.g. explanatory video with pop-up questions) and design a test including automatic evaluation. The elements should not be generated as empty structures but filled with suitable content. In addition, the processing sequence should be defined so that students can be guided through the learning unit. All learning units were created within the same Moodle course so that the pre-service teachers could see the activities created by the other participants of the training course and use them as inspiration and for support. Besides, an exchange between the participants was possible in an implemented forum.

The pre-service teachers had one week to develop the learning unit in the self-study phase. Afterward, the chances and challenges that they experienced while creating the learning unit were discussed in an online-meeting. Furthermore, the pre-service teachers were divided into groups to test the learning units of other participants (as fictive students) and give mutual feedback according to several defined criteria.

With regard to the specific conditions of the online training course and the *Tripartite Model of Attitude Structure*, the research question as described above can be divided into the following sub-questions:

(1a): Which **emotions** can be identified **before** the training?
(1b): Which **behavioral intentions** can be identified **before** the training?
(1c): Which **perceptions, knowledge, and beliefs** can be identified **before** the training?
(2a): Which **emotions** can be identified **after** the training?
(2b): Which **behavioral intentions** can be identified **after** the training?
(2c): Which **perceptions, knowledge, and beliefs** can be identified **after** the training?

In order to investigate the attitudes towards the use of online learning platforms and especially their changes due to the training, a research design consisting of two open questionnaires (pre and post) is chosen. The pre-questionnaire was completed by the pre-service teachers before the training. It consists of a total of nine questions addressing different aspects of attitudes towards online learning platforms:

1. What is your experience with online platforms for learning mathematics at school? Which platforms do you know?
2. What chances do you see in the use of online platforms for learning mathematics at school? Explain your answer!
3. What challenges do you see in the use of online platforms for learning mathematics at school? Explain your answer!
4. For which purposes would you use online platforms for learning mathematics at school? Explain your answer and give examples!
5. For which purposes would you not use online platforms for learning mathematics at school? Explain your answer!
6. How does the use of online platforms change the learning of mathematics at school? Explain your answer!
7. Do you associate certain emotions with the use of online platforms for learning mathematics at school?
8. Give a final conclusion on online platforms for learning mathematics at school!

The post-questionnaire was completed by the pre-service teachers after the training on online learning platforms. It contains the same questions as the pre-questionnaire, but they were specifically asked about the changes due to the training and they should refer to their answers in the first questionnaire (e.g. 1. What experience have you gained with online platforms for learning mathematics at school through the training?). A total of 14 pre- and 14 post-questionnaires were completed and could be included in the analysis. The questionnaires were provided as an editable word document via Moodle.

**Fig. 2:**
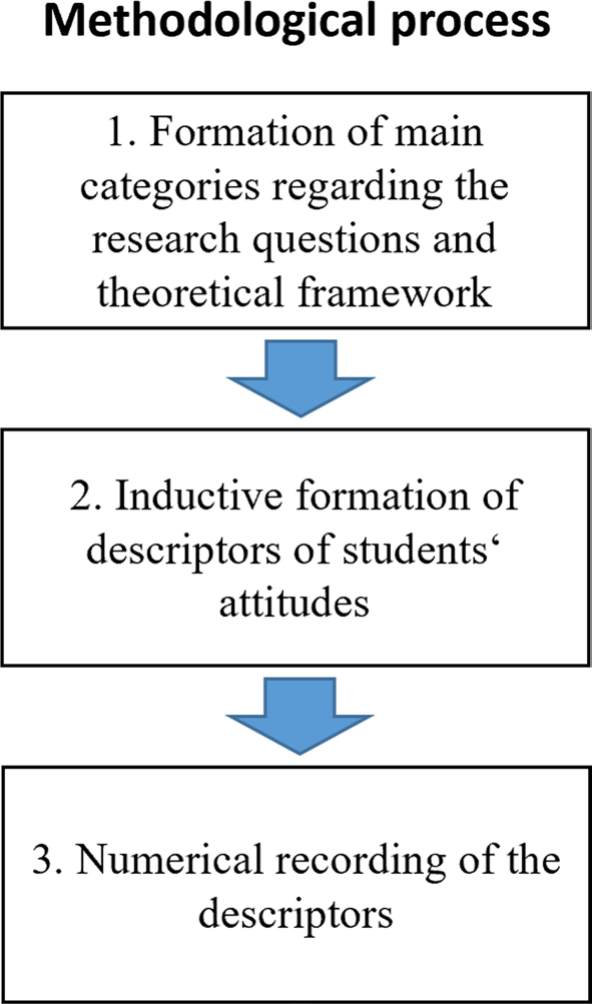
Flowchart of the methodical process

The data material was analyzed using the method of qualitative content analysis according to Mayring (2000). This is a comparatively structured form of coding statements, which was originally developed in Social Sciences. The process of data analysis took place in three separate steps (see also Fig. 2):

In the *first step*, a system of main categories was formed by deductive derivation from the research questions and the underlying *Tripartite Model of Attitude Structure*. This system of categories comprised of six main categories:

- *A1: Affective Component before the Training* Statements of the pre-questionnaire that refer to emotions in relation to online learning platforms.
- *B1: Behavioral Component before the Training* Statements of the pre-questionnaire that refer to intended behavior in relation to online learning platforms.
- *C1: Cognitive Component before the Training* Statements of the pre-questionnaire that refer to beliefs and knowledge in relation to online learning platforms.
- *A2: Changes of the Affective Component due to the Training* Statements of the post-questionnaire that refer to the change of emotions in relation to online learning platforms.
- *B2: Changes of the Behavioral Component due to the Training* Statements of the post-questionnaire that refer to the change of intended behavior in relation to online learning platforms.
- *C2: Changes of the Cognitive Component due to the Training* Statements of the post-questionnaire that refer to the change of beliefs and knowledge in relation to online learning platforms.

In the *second step*, the statements were coded by inductively forming descriptors for the main categories, according to the method of the structuring qualitative content analysis (Mayring, 2000). For this purpose, the data material was first described in detail and the unit of analysis was determined. In the present case, the analyzed data included the completed pre- and post-questionnaires of 14 participants. The unit of analysis, the smallest analyzed part, is each statement of the participants that refers to digital technologies in education, especially online learning platforms. Afterward, the statements of the pre-service teachers were paraphrased, reoccurring statements were combined and finally described in a system of categories. It contains the definition of a descriptor and an example from the data material (in the tables in the appendix only the names of the descriptors and an example from the data material are provided, as these are mainly self-explanatory).

The *third step* was the determination of the number of participants for which the descriptors could be identified. Accordingly, a value between 2 and 14 was assigned to each category. The frequencies are only intended to provide a rough overview of the distribution and do not allow any statistical statements to be made due to the small number of participants.

### Results and Interpretation

The analysis of the data material revealed a total of 51 descriptors for affective, behavioral, and cognitive aspects related to online platforms for the learning of mathematics. Beliefs about online learning platforms as part of the cognitive component represent the majority of the identified descriptors—emotions and behavioral intentions could be determined in a much less differentiated way. The identified aspects are explained in the following section regarding the respective main category.

First of all, the descriptors formed on the basis of the pre-questionnaires are presented in order to reflect the pre-service teachers' attitude towards online learning platforms before the training. The affective component comprises two descriptors, which represent emotions regarding the use of online platforms (see Table 1, Appendix). Three pre-service teachers report that they feel insecure and overloaded due to a lack of preparation regarding the use of such platforms (A1-1). Other statements show more positive emotions in relation to online learning platforms. In particular, two pre-service teachers say that they are pleased about the relief in class (A1-2).

A total of eight descriptors can be assigned to the behavioral component (see Table 2, Appendix). Six of these are descriptors for scenarios in which pre-service teachers would use online platforms. Five participants state that they would like to use them in addition to classroom teaching (B1-1). Four of them would use the online platform for the practice of topics (B1-2), for individual support and differentiation (B1-3) as well as for the visualization of mathematical topics (B1-4). Three students intend to integrate learning videos (B1-5) and another two would use it to provide material (B1-6).

Two of the descriptors of the behavioral component refer to scenarios in which the participants would explicitly not use an online platform. Six participants would not use the platform to introduce a topic (B1-7). Five of the participants also do not want to teach purely online, but only in combination with face-to-face teaching (B1-8).

#### Statements Before the Online Training

Most of the statements of the pre-service teachers in the pre-questionnaires can be assigned to the cognitive component. A total of 29 descriptors can be formed which represent the participants' personal evaluations of their experiences and competences, of the opportunities, challenges and general developments with regard to online platforms for learning mathematics (see Table 3, Appendix).

Three of the descriptors relate to the experiences and competences of the participants. All 14 pre-service teachers have experience in using Moodle from a student perspective at university (C1-1). Eight participants explicitly state that they have no experience with online platforms at school (C1-2). Three participants have had some experience as students at school (C1-3).

Thirteen descriptors have been formed on the opportunities offered by online platforms. All 14 participants indicate that online platforms can enrich teaching (C1-4). Eight participants assume an increased motivation among students (C1-5) and another seven participants state that visualizations are particularly helpful (C1-6). Four participants state that students can determine the time and pace of the learning process themselves (C1-7). Three participants describe it as a chance that the material and the submissions of the students are saved in an orderly way (C1-8), that networking between students and the teacher is easily possible (C1-9), that individualization of the learning process can be realized more easily (C1-10), that the students learn to work independently (C1-11), that there is a relief in teaching (C1-12) and that the platform leads to a saving in time (C1-13). Finally, the possibility of promoting the media competence of the students (C1-14), the easier continuation of lessons in case of crises with school closures (C1-15) as well as the automatization of the evaluation of tests (B1-16) are mentioned as an advantage of two participants each.

A total of nine descriptors deals with the challenges of the use of online platforms. Twelve participants see a challenge in the media competences of teachers (C1-17), six in the media competences of students (C1-18). Another six participants consider an increased time effort for the students (C1-19) and four participants emphasize that the medium should not become an aim in itself (C1-20). The technical equipment of the students (C1-21), as well as their lack of motivation (C1-22), are seen as a challenge by three participants. Another three participants assume that publishing solutions might motivate students to overuse them (C1-23). Two participants see a problem in the equipment of the schools (C1-24) as well as in the insufficient possibility to give feedback in real-time (C1-25).

Four descriptors of the cognitive component refer to neutral statements on general developments. Four participants assume that online platforms lead to more autonomous work (C1-26), three participants think that there is less intervention by the teacher (C1-27). Two participants claim that learning becomes more decentralized and distributed to other locations (C1-28) and that teaching is modernized through learning platforms (C1-29).

#### Statements After the Online Training

The statements of the post-questionnaire were analyzed with regard to the changes in attitudes towards online learning platforms due to the training. It was possible to form 29 descriptors that can be assigned to the affective, behavioral, and cognitive components.

Two of these descriptors can be assigned to the affective component, as they express emotions in relation to online platforms (see Table 4, Appendix). Three people reported that they are pleased by the variety of possibilities offered by online platforms (A2-1). In contrast, two participants are frustrated because of problems with the software (A2-2).

Three descriptors can be assigned to the behavioral component, which describes intended and non-intended usage scenarios (see Table 5, Appendix). Intended scenarios include the use of online teaching, for example, if online teaching has to take place due to a pandemic (B2-1), which was mentioned by three participants. With regard to non-intended scenarios, three participants revise their original statement from the pre-questionnaire and say that they would now also use online platforms to introduce a topic (B2-2). In contrast to the statements they made before the training course, two participants now claim that they do not want to conduct pure online teaching with platforms (B2-3).

Based on the post-questionnaire, seven descriptors can be assigned to the cognitive component (see Table 6, Appendix). First of all, 13 participants indicate that they have gained experience in creating online platforms through the training and were able to develop corresponding competences (C2-1). With regard to the chances of online platforms, some participants also mentioned new aspects. Three of them now see the possibility of guiding their future students through the learning environment by programmed prerequisites (C2-2) as an opportunity after the training. The simple continuation of the lessons in case of crises with school closures (C2-3) as well as the automatization of feedback (C2-4) are seen as a chance by two participants.

Changes in the challenges mentioned by the participants can also be identified. Seven participants state that online platforms for learning are associated with an increased preparation effort (C2-5). Three persons also state that the software is partly not user-friendly (C2-6) and is not completely developed yet (C2-7).

### Discussion

The empirical study identified many aspects of pre-service teachers' attitudes towards the online platform Moodle for learning mathematics. With the help of the pre-questionnaire, the affective, behavioral, and cognitive aspects of the attitudes were investigated in a differentiated way. With reference to the first research question

*Which emotions can be identified before the training?*

two descriptors were formed, which showed certain insecurity of the pre-service teachers, but also positive emotions such as pleasure. In general, however, not so many emotions could be identified. One reason for this may be that the participants had little or no previous experience with online platforms so that the topic is not that much emotionally charged. In addition, only explicit statements on emotions were included. Finally, a written questionnaire format is probably of only limited use in capturing emotions.

The second research question

*Which behavioral intentions can be identified before the training?*

can be answered in a more differentiated way based on the data material. Many participants stated that they only want to use learning platforms in addition to and not as a substitute for classroom teaching. Furthermore, statements are made about the phases in which the platform should be used. All in all, it can be seen that the participants consider the use of the platform for practicing a topic to be useful, but do not want to introduce a topic with the online platform. The primary application scenarios mentioned in the questionnaires are differentiation and individual support, the visualization of mathematical issues and integration of learning videos.

The majority of descriptors can be formed with regard to the third research question:

*Which perceptions, knowledge, and beliefs can be identified before the training?*

The previous experience of the participants was especially limited to using Moodle as a university student. Only one person has some experience with the use of learning platforms from the teacher's perspective. Nevertheless, a variety of different beliefs about chances and challenges as well as the general developments became apparent. All the pre-service teachers surveyed see online learning platforms as an enrichment for teaching. This positive image is reflected in the more concrete beliefs about the chances of online platforms. Many participants suspect an increased intrinsic motivation of the students in the classroom and see a great advantage in the good possibilities of visualizing facts. The pre-service teachers also find it important to be able to determine the time and pace of the learning process on their own. Many other opportunities are mentioned by the participants, addressing various aspects of online platforms.

In addition to the positively attested beliefs, the pre-service teachers also recognize many challenges of online platforms for learning mathematics. Almost all participants mention the media competences of teachers and students at this point. In addition, many participants mention the increased time of the students required for learning, the technical equipment of the students, and the lack of the students’ self-motivation. The participants also emphasize that the platform has to be filled with useful content and that the publishing of solutions could lead to misuse by the students in class. The pre-service teachers name a variety of other challenges that will not be repeated here.

Some neutrally connoted statements about general developments are also made by the participants. They describe that there is a decentralization of teaching in which students mainly work independently and the teacher has less influence on the learning process of the students.

The three other research questions focus on the changes in attitudes due to the training and can be answered based on the pre-service teachers' answers in the post-questionnaire. For the fourth research question

*Which emotions can be identified after the training?*

the pre-service teachers mentioned some additional emotions, and descriptors that have already been identified in the pre-questionnaire could partly be identified for other participants in this questionnaire as well. Pleasure about the variety of possibilities of the software and the new way of working as well as frustration due to problems in using the software could be identified. In the post-questionnaire, however, as in the pre-questionnaire, only a few explicit statements about emotions could be found.

The fifth research question

*Which behavioral intentions can be identified after the training?*

also resulted in some additional descriptors. In particular, it was found that some statements from the pre-questionnaire were explicitly withdrawn. Three persons, who previously thought that the introduction of a topic via online platforms could not be carried out in a meaningful way, now think that the platform is also suitable for this purpose. Some additional statements about the behavior could be identified, but will not be described in detail here.

For the sixth research question

*Which perceptions, knowledge, and beliefs can be identified after the training?*

many descriptors were identified so that the situation can be reconstructed in a comparatively differentiated way. Almost all participants stated that they have gained experience and competences in the creation of content on online platforms. This leads to a multitude of additional beliefs regarding the opportunities and challenges of the digital medium. Especially the guiding of the student's learning process by programming prerequisites, the automatization of feedback as well as the easy continuation of teaching in times of crisis are mentioned as new chances. Many participants see the increased preparatory effort as well as the lack of user-friendliness and sophistication of the software as a challenge after the training.

All in all, it could be seen that the participants of the course had already mentioned many and partly very different aspects regarding online learning platforms before the training was carried out, although they had little experience with the medium until then. In general, the participants’ attitudes seem to be positive, but challenges were considered as well. After the training, almost all participants stated that they had gained experience and skills. This was associated with a multitude of new beliefs, behavioral intentions, and emotions. Thus, the training led the pre-service teachers to deal with the topic in-depth and to develop a more differentiated and reflected attitude. The general attitude towards online platforms for learning mathematics remains positive. However, it should be mentioned here that all participants have become acquainted with Moodle in their previous semesters form the learner’s perspective, because Moodle is commonly used in teacher education at the University of Siegen and that the participants have had a complete online semester at university during the training course on online learning platforms. Both facts could have had an influence on their attitude towards online learning platforms as well.

## Conclusion

As shown in Sect. 2.2, some research findings about online learning platforms have already been obtained in empirical studies. Many of these studies show that the systems can have a (positive) impact on learning in schools and change learning processes (e.g., see Henríquez et al., 2018; Pérez-Álvarez et al., 2018; Davis et al., 2018; Zhao et al., 2018; Paechter et al., 2013). Several factors can be distinguished for a successful use of this technology, including the role of the teacher (see Moser & Petko, 2010a, b) and his/her attitudes. Research on attitudes towards digital technologies in the classroom (in our case on the example of mathematics education) is already comparatively well developed (see Sect. 3.3). Various empirical studies have been able to show that positive and reflective attitudes toward digital technology are related to meaningful use of technologies in the classroom (e.g., Li et al. 2019, Van Aalderen-Smeets et al. 2012, Nordlöf et al. 2019). Such attitudes not only exist in general, but can also relate to specific digital technologies. With respect to online learning platforms, there is a research gap, which this paper addresses.

Regarding the main research question *“How do pre-service teachers reflect their attitudes towards online learning platforms in mathematics class at school and how do their reflections change after an online training course on this topic?”*, in summary, it can be said that the pre-service teachers describe their attitude towards the use of online learning platforms in detail even before the training in the seminar and they name various aspects, especially in the field of the behavior and cognitive components of their attitude.

It is interesting to note that the pre-service teachers only express a few emotions explicitly in the pre-questionnaire, which can be a result of the framework of the study, especially the written and explicit questionnaires. After the training, there are not only positive emotions like pleasure but also frustration caused by usage problems. Therefore, when conducting training on the use of learning platforms, it is important to be aware that there may be problems that affect the attitudes of the participants in a negative way. This might be prevented by introducing phases of reflection on the errors and problems that occur during the training and documenting (e.g. in FAQs). In this context, it should be added that Moodle offers a FAQ for beginners or a forum for solving technical problems.

The behavioral intentions identified before the training focus on the enrichment of traditional face-to-face-teaching and learning. Pre-service teachers do not intend to use online learning platforms as a substitute for face-to-face teaching. They would mainly use learning platforms for differentiation, individualization of the learning process, and visualization. The main change in this component after the training is that they have the opinion that the platform is suitable for the introduction of a new topic. The reason for this could be that they see advantages in special functions of the platform (such as the repeated playing of videos or access to differentiated and individualized material).

Most of the pre-service teachers’ statements, both before and after the training, have been associated with the cognitive component of their attitude. Although they have almost no previous experience and therefore no sophisticated skills and abilities in dealing with learning platforms, the pre-service teachers designate concrete beliefs about the chances and challenges of online platforms before the training. Two aspects appeared particularly frequently, namely the increase of the intrinsic motivation of the students in class and the opportunities for visualization. Furthermore, they mentioned the decentralization of the learning process that can be assigned to the change and transformation of the learning process in general. Online learning platforms enable self-determined learning because learning can take place at any time and any place (mobile-learning). Pre-service teachers are also aware that the extent and nature of the influence of the teacher on the student's learning process is changing. As already mentioned in the literature review, the teacher becomes a learning facilitator instead of a knowledge transmitter. In this context, the pre-service teachers see challenges in media competences of teachers and students, existing technical equipment, and the lack of motivation of students. Even before the training, they have the opinion that only the content makes an online learning platform a suitable tool for teaching. This statement can be set to the postulate of Petko (2010a, p. 16, authors’ translation): “*It must not be a matter of adapting didactic to the possibilities offered by technology, but rather the other way round.”* After the training, all pre-service teachers notice that they have gained experience and skills. Therefore, we can assume that the training has a positive effect on the pre-service teachers’ knowledge of online platforms (in particular *Moodle*), but also on their attitudes towards the use of online learning platforms in mathematics. Their attitudes are now even more differentiated and reflected and they are able to clearly define and justify changes and challenges. This can be an important point for the later use of this tool in the classroom.

The original contribution of the paper are the descriptors, that reflect important aspects of pre-service teachers' attitudes towards online platforms using Moodle as an example. Some of the identified descriptors could be specific for online learning platforms, while others have also been found in different and general contexts in empirical studies (e.g. the increased time spent by teachers or learners as a challenge in the study of Thurm, 2017). The present study also shows that an online training can have an impact on attitudes and that students reflect on them in a different, perhaps sharpened, way after the training.

The identified emotions, behavioral intentions, and beliefs enrich the research in the field of attitudes towards digital technologies, but in particular towards online learning platforms. With the chosen method and the general conditions, it is not possible to obtain generally valid results. This is firstly due to the small number of participants in the study, and secondly due to the reflection questionnaire, which only captures explicit attitudes that the participants are aware of. Hence, the objective of the study was to gain initial insights in the area of attitudes and online learning platforms at a qualitative level, that can form the basis of several follow-up studies on the basis of the identified descriptors. Furthermore, in times of Covid-19, there is a growing requirement for methods of meaningful analysis of key elements of implementing online platforms in the mathematics classroom. The findings of this case study show that a specialized online training on online platforms as an advanced digital tool for distance-learning can improve the impact of teacher education at university. This is a relevant result for the research on teacher training and online teacher training in particular. However, the results might be transferable to teachers who already teach at school, as they have also attitudes towards specific digital technologies. The paper shows the importance of pre- and in-service teacher training for the development of differentiated and reflected attitudes towards new technologies.

## Appendix

See Tables

**Table 1:** Descriptors of the affective component before the training

Affective Component before the Training
A1-1*: Insecurity and overload due to lack of preparation* (3) (“I feel uncertain because we have not been prepared for the use of the online platforms at all.”)
A1-2: *Pleasure, about relief in class* (2) (“[…] but I am equally pleased, as they provide some relief in the classroom.”)

1,

**Table 2:** Descriptors of the behavioral component before the training

Behavioral Component before the Training:
*Use intended:*
B1-1: *In addition to classroom teaching* (5) (“However, I would use it in addition to attendance teaching […].”)
B1-2: *Practicing and consolidation of a topic* (4) (“I would use online platforms to further practice already known topics.”)
B1-3: *Differentiation and individual support* (4) (“[…] as a supplement to the teaching in order to support the students specifically.”)
B1-4: *Visualization of mathematical issues* (4) (“I would use online platforms mainly for visualization […].”)
B1-5: *Integration of learning videos* (3) (“I also think that learning videos are a useful idea […].”)
B1-6: *Provision of material* (2) (“I would upload blackboard pictures and other teaching material […].”)
*Use not intended:*
B1-7: *Not for the introduction of a topic* (6) (“I would avoid using online platforms at the beginning of a new topic.”)
B1-8: *Not as a pure online teaching* (5) (“I would not use online teaching as a substitute for normal teaching […].”)

2,

**Table 3:** Descriptors of the cognitive component before the training

Cognitive Component before the Training:
*Experiences and competences:*
C1-1: *Experience as student at university* (14) (“From the university I know Moodle.”)
C1-2: *No experience as student or teacher at school* (8) (“I don’t have any experience with online platforms at school.”)
C1-3: *Some experience as student at school* (3) (“I know that I had to work with an online platform in my school days in the subject pedagogy, but I can't remember the name of it.”)
*Chances:*
C1-4: *Enrichment of teaching* (14) (“All in all, I believe that online platforms can enrich mathematics teaching.”)
C1-5: *Increased intrinsic motivation of students* (8) (“In my opinion, students can be motivated by using online platforms […].”)
C1-6: *Visualization of connections is possible* (7) (“Such platforms can be used to visualize complex content well.”)
C1-7: *Students can determine the time and pace of learning themselves* (4) (“I see the chance that the students can manage their learning time better.”)
C1-8: *Materials and student solutions are stored in an ordered manner* (3) (“Students can submit their exercises and they are be ordered and bundled.”)
C1-9: *Better networking of teachers and learners* (3) (“By using online learning platforms, a better networking of teachers and learners can be realized.”)
C1-10: *Differentiation through individualization of the learning process* (3) (“In general, a greater individualization of the learning process can take place.”)
C1-11: *Encourage students to work independently* (3) (“I see it as a chance that the students learn to work independently.”)
C1-12: *Relief during teaching* (3) (“I can imagine that the digital medium makes teaching easier […].”)
C1-13: *Time saving with the platform* (3) (“The platform could result in time savings in teaching.”)
C1-14: *Promoting students' media competences* (2) (“[…] since in addition to mathematical competences, media competence is also promoted and demanded.”)
C1-15: *Simplified continuation of work in crises with school closure* (2) (“In the light of the current exceptional situation, an existing online platform is also good because it allows teaching to continue without improvisation.”)
C1-16: *Automatic evaluation of tasks* (2) (“These would be used for homework, which would provide the students with a solution immediately after submission […].””)
*Challenges:*
C1-17: *Media competences of teachers* (12) (“The biggest challenge for teachers is to have enough media competence themselves and to always stay "up-to-date".”)
C1-18: *Media competences of students* (6) (“Especially for students who have little experience with such platforms, it can be very difficult to handle them.”)
C1-19: *Increased time spent by students using the platform* (6) (“Time is also needed to discover new things on the platforms.”)
C1-20: *Medium should not become an aim in itself* (4) (“The content should remain in the foreground, not the medium.”)
C1-21: *Technical equipment of the students* (3) (“Not every child has access to a computer.”)
C1-22: *Lack of self-motivation of students* (3) (“[…] because the effective use of online platforms for learning requires a certain degree of self-motivation and personal impetus in learning.”)
C1-23: *Published solutions lead to improper handling* (3) (“As the solutions are often directly accessible, some students do not solve the tasks independently […].”)
C1-24: *Technical equipment of the school* (2) (“A challenge is definitely the feasibility, as a suitable platform must first be found, financed, set up, operated and disseminated.”)
C1-25: *Feedback in real time difficult to realize* (2) (“I see it as a challenge that individual questions often cannot be answered in real time.”)
*Neutral statements on the general developments:*
C1-26: *More independent work* (4) (“The students can work more freely and above all independently […]”)
C1-27: *Less intervention by the teacher* (3) (“Teachers can create online learning environments, but they cannot intervene in the learning process of the students to the same extent as if they were present with the students […].”)
C1-28: *Learning becomes more decentralized* (2) (“In particular, learning is becoming more decentralized, it can be "taken home" and it is easier to learn from home or elsewhere.”)
C1-29: *Teaching becomes more modern* (2) (“Mathematics teaching becomes […] more modern […]”)

3,

**Table 4:** Descriptors of the changes of the affective component due to the training

Changes of the Affective Component due to the Training:
A2-1: *Pleasure about the many possibilities* (3) (“It's great that you can create so many different tasks with it.”)
A2-2: *Frustration due to mistakes and problems* (2) (“It caused negative emotions in me that there were errors and problems […] that could not be solved […].”)

4,

**Table 5:** Descriptors of the changes of the behavioral component due to the training

Changes of the Behavioral Component due to the Training:
*Use intended:*
B2-1: *Online platform instead of emails, when teaching has to be conducted online* (2) (“Besides, I would use them instead of emails when the lessons have to take place online.”)
*Use not intended:*
B2-2: *Statement revised: also good for the introduction of a topic* (3) (“Previously, I thought it would be a bad idea to introduce new topics with the platform. Because of all the possibilities of presentation I reject this estimation […].”)
B2-3: *Not a pure online teaching* (2) (“But I am not in favour of solely teaching online at school […].”)

5 and

**Table 6:** Descriptors of the changes of the cognitive component due to the training

Changes of the Cognitive Component due to the Training:
*Experiences and competences:*
C2-1: *Experience gained with the creation of activities on online platforms* (13) (“I have gained experience in creating online offers for students on online platforms.”)
*Chances:*
C2-2: *Guiding through the tasks by programmed requirements* (3) (“Furthermore, I think it is great that the tool with the requirements for testing is very useful. The students must have achieved tasks, maybe even with a certain score, to take the test.”)
C2-3: *Simplified continuation of work in crises with school closure* (2) (“What I also noticed with regard to the current situation is that it is much easier to continue teaching in case of a pandemic or other events that prevent attendance.”)
C2-4: *Automatic preparation of feedback* (2) (“Automating feedback is a good way for students to give an initial but impersonal assessment of their performance.”)
*Challenges:*
C2-5: *Increased preparatory effort* (7) (“Although the handling will be easier in the long-term, I believe that it is still more effort to prepare a digital lesson.”)
C2-6: *Software partial not user-friendly* (3) (“[…] because the program is designed a bit confusing for the teacher.”)
C2-7: *Software partially not fully developed* (3) (“But dealing with it has shown me that the platforms are not yet fully developed in many respects.”)

6
